# Crystal structure of 2-(4-chloro-3-fluoro­phen­yl)-1*H*-benzimidazole

**DOI:** 10.1107/S2056989015008683

**Published:** 2015-05-09

**Authors:** M. S. Krishnamurthy, Noor Shahina Begum

**Affiliations:** a Department of Studies in Chemistry, Central College Campus, Bangalore University, Bangalore 560 001, Karnataka, India

**Keywords:** crystal structure, benzimidazole, fluorine-containing compound, hydrogen bonding, C—H⋯π inter­actions, π–π inter­actions

## Abstract

In the title compound, C_13_H_8_ClFN_2_, the dihedral angle between the plane of the benzimidazole ring system (r.m.s. deviation = 0.022 Å) and the benzene ring is 26.90 (8)°. The F atom at the *meta* position of the benzene ring is disordered over two sites in a 0.843 (4):0.157 (4) ratio. In the crystal, mol­ecules are linked by N—H⋯N hydrogen bonds, forming infinite *C*(4) chains propagating along [010]. In addition, weak C—H⋯π and π–π inter­actions [shortest centroid–centroid separation = 3.6838 (12) Å] are observed, which link the chains into a three-dimensional network.

## Related literature   

For therapeutic and medicinal properties of benzimidazole derivatives, see: Ozden *et al.* (2004 ▸); Easmon *et al.* (2001 ▸); Thakurdesai *et al.* (2007 ▸); Ansari & Lal (2009 ▸). For the bioactivity of fluorine-containing compounds, see: Ulrich (2004 ▸). For related structures, see: Fathima *et al.* (2013 ▸); Jian *et al.* (2006 ▸); Krishnamurthy & Begum (2014 ▸); Krishnamurthy *et al.* (2013 ▸); Rashid *et al.* 2007 ▸); Jayamoorthy *et al.* (2012 ▸); Yoon *et al.* (2012 ▸). Positional disorder is common in many organic compounds containing fluorine in either the *ortho* or *meta* position, see: Chopra & Guru Row (2008 ▸); Nayak *et al.* (2011 ▸). For normal C—F bond lengths, see: Zhang *et al.* (1998 ▸).
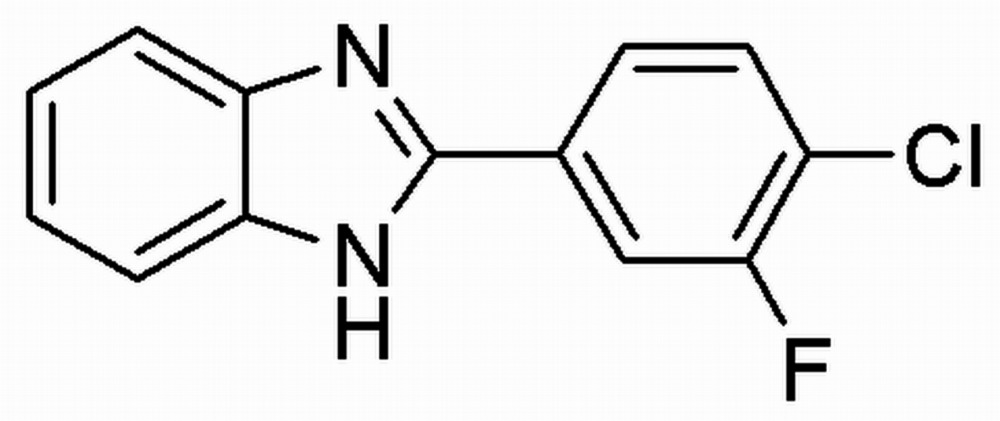


## Experimental   

### Crystal data  

C_13_H_8_ClFN_2_
*M* *_r_* = 246.66
Orthorhombic, 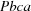

*a* = 9.2302 (4) Å
*b* = 9.8500 (4) Å
*c* = 23.1347 (9) Å
*V* = 2103.35 (15) Å^3^
*Z* = 8
Mo *K*α radiation
μ = 0.35 mm^−1^
*T* = 100 K
0.18 × 0.16 × 0.16 mm

### Data collection  

Bruker SMART APEX CCD diffractometer
Absorption correction: multi-scan (*SADABS*; Bruker, 1998 ▸) *T*_min_ = 0.940, *T*_max_ = 0.946
23308 measured reflections
1844 independent reflections
1606 reflections with *I* > 2σ(*I*)
*R*_int_ = 0.043

### Refinement  

*R*[*F* ^2^ > 2σ(*F* ^2^)] = 0.038
*wR*(*F* ^2^) = 0.102
*S* = 0.95
1844 reflections
164 parameters
H-atom parameters constrained
Δρ_max_ = 0.56 e Å^−3^
Δρ_min_ = −0.32 e Å^−3^

### 

Data collection: *SMART* (Bruker, 1998 ▸); cell refinement: *SAINT* (Bruker, 1998 ▸); data reduction: *SAINT*; program(s) used to solve structure: *SHELXS97* (Sheldrick, 2008 ▸); program(s) used to refine structure: *SHELXL97* (Sheldrick, 2008 ▸); molecular graphics: *ORTEP-3 for Windows* (Farrugia, 2012 ▸) and *CAMERON* (Watkin *et al.*, 1996 ▸); software used to prepare material for publication: *WinGX* (Farrugia, 2012 ▸).

## Supplementary Material

Crystal structure: contains datablock(s) global, I. DOI: 10.1107/S2056989015008683/hb7406sup1.cif

Structure factors: contains datablock(s) I. DOI: 10.1107/S2056989015008683/hb7406Isup2.hkl

Supporting information file. DOI: 10.1107/S2056989015008683/hb7406Isup3.cml

**Fig. 1.**
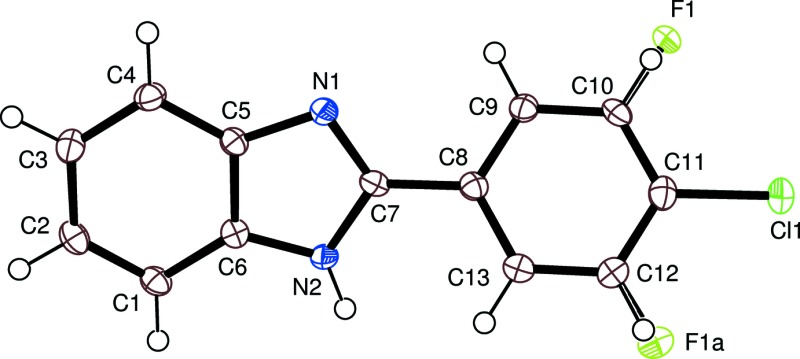
The molecular structure of the title compound with displacement ellipsoids drawn at the 50% probability level.

. DOI: 10.1107/S2056989015008683/hb7406fig1.tif
The mol­ecular structure of the title compound with displacement ellipsoids drawn at the 50% probability level.

**Fig. 2.**
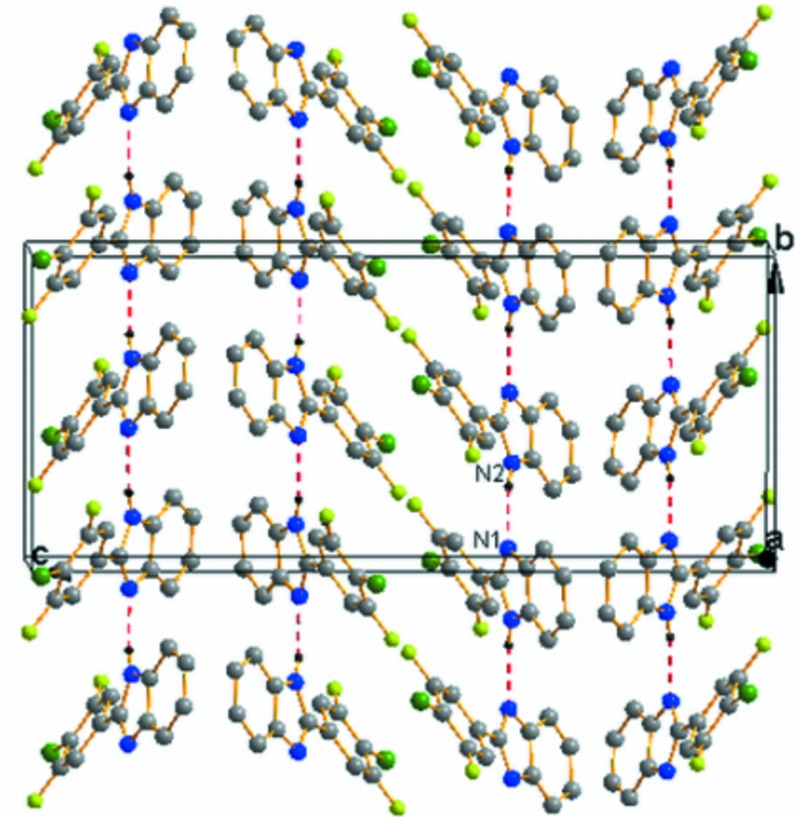
Unit cell packing of the title compound showing N—H···N interactions with dotted lines. H-atoms not involved in hydrogen bonding have been excluded.

. DOI: 10.1107/S2056989015008683/hb7406fig2.tif
Unit cell packing of the title compound showing N—H⋯N inter­actions with dotted lines. H-atoms not involved in hydrogen bonding have been excluded.

**Fig. 3.**
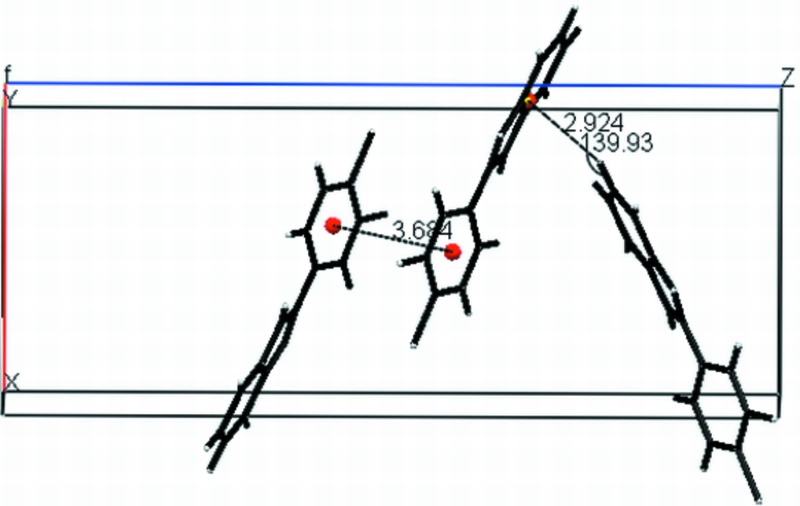
Unit cell packing showing C—H···π and π–π interactions with dotted lines.

. DOI: 10.1107/S2056989015008683/hb7406fig3.tif
Unit cell packing showing C—H⋯π and π–π inter­actions with dotted lines.

CCDC reference: 1063160

Additional supporting information:  crystallographic information; 3D view; checkCIF report

## Figures and Tables

**Table 1:** Hydrogen-bond geometry (, ) *Cg* is the centroid of the N1/C5/C6/N2/C7 ring.

*D*H*A*	*D*H	H*A*	*D* *A*	*D*H*A*
N2H2N1^i^	0.88	2.06	2.924(1)	166
C3H3*Cg* ^ii^	0.95	2.92	3.700(3)	140

Symmetry codes: (i) 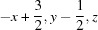; (ii) 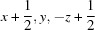.

## supplementary crystallographic information

### S1. Comment

Benzimidazole and their derivatives are known to exhibit a wide variety of pharmacological properties. Benzimidazole is an important pharmacophore and a privileged structure in medicinal chemistry encompassing a diverse range of biological activities such as antibacterial (Ozden *et al.*, 2004), anticancer (Easmon *et al.*, 2001), anti-HIV and anti-inflammatory (Ansari & Lal 2009; Thakurdesai *et al.*, 2007). Benzimidazole and its derivatives can also be used as ligands in the field of coordination Chemistry. In addition, compounds which contain fluorine have special bioactivity (Ulrich, 2004). Herein, we report the crystal structure of the title compound. The molecular structure of the title compound C_13_H_8_ClFN_2_ is shown in Fig. 1. It is the fluoro-analogue of our previously reported compounds (Fathima *et al.*, 2013; Krishnamurthy *et al.*, 2013; Krishnamurthy & Begum., 2014). The benzimidazole system is essentially planar, with a dihedral angle of 2.251 (6)° between the planes of the benzene ring and its fused imidazole ring. The whole molecule is nonplanar; the dihedral angle between the benzimidazole ring and the benzene ring is 26.898 (1)°. This value is slightly lower than that observed in related compounds (Jian *et al.*, 2006; Krishnamurthy & Begum, 2014). It was observed that the fluorine atom at the *meta* position is disordered over two sites, the major occupancy refining to 0.843 (4) and minor occupancy is 0.157 (4). In fact, the C—F bond length associated with the major occupancy fluorine is almost close to the standard C—F bond length (1.345 Å) while the minor occupancy fluorine has a bond length lying between normal value of C—H and C—F bond (Zhang *et al.*, 1998). This type of positional disorder is common in many organic compounds containing fluorine in either the *ortho* or *meta* position (Chopra *et al.*, 2008; Nayak *et al.*, 2011). In the crystal structure, the molecules are linked by intermolecular N2—H2···N1 hydrogen bonds to form infinite chains parallel to [010] (Table. 1; Fig. 2). In addition, a weak C—H···π interaction of the type C3—H3···*Cg* (*Cg* being the centroid of the benzimidazole ring) link chains into layers parallel to [001] and π–π stacking interactions with a centroid—centroid distance of 3.684 (10) Å connect these layers into a three-dimensional network (Fig. 3).

### S2. Experimental

The title compound was synthesized by refluxing 3-fluoro, 4-chlorobenzaldehyde (20 mmol, 0.28 g) and *o*-phenyldiamine (20 mmol, 0.22 g) in benzene (3.0 ml) for 6 hrs on a water bath. The reaction mixture was cooled. The solid separated, was filtered and dried (yield = 0.36 g (76%) and M.P. = 526 K). Yellow blocks were obtained by slow evaporation of an ethyl acetate solution.

### S3. Refinement

The H atoms were placed in calculated positions and refined in a riding model approximation with C—H= 0.93 Å, N—H=0.86 Å and with *U*_iso_(H) = 1.2*U*_eq_(N/C).

### Crystal data

**Table:**

C_13_H_8_ClFN_2_	*F*(000) = 1008
*M**_r_* = 246.66	*D*_x_ = 1.558 Mg m^−^^3^
Orthorhombic, *P**b**c**a*	Mo *K*α radiation, λ = 0.71073 Å
Hall symbol: -P 2ac 2ab	Cell parameters from 1844 reflections
*a* = 9.2302 (4) Å	θ = 2.8–25.0°
*b* = 9.8500 (4) Å	µ = 0.35 mm^−^^1^
*c* = 23.1347 (9) Å	*T* = 100 K
*V* = 2103.35 (15) Å^3^	Block, yellow
*Z* = 8	0.18 × 0.16 × 0.16 mm

### Data collection

**Table:**

Bruker SMART APEX CCD diffractometer	1844 independent reflections
Radiation source: fine-focus sealed tube	1606 reflections with *I* > 2σ(*I*)
Graphite monochromator	*R*_int_ = 0.043
ω scans	θ_max_ = 25.0°, θ_min_ = 2.8°
Absorption correction: multi-scan (*SADABS*; Bruker, 1998)	*h* = −10→10
*T*_min_ = 0.940, *T*_max_ = 0.946	*k* = −11→11
23308 measured reflections	*l* = −27→27

### Refinement

**Table:**

Refinement on *F*^2^	Primary atom site location: structure-invariant direct methods
Least-squares matrix: full	Secondary atom site location: difference Fourier map
*R*[*F*^2^ > 2σ(*F*^2^)] = 0.038	Hydrogen site location: inferred from neighbouring sites
*wR*(*F*^2^) = 0.102	H-atom parameters constrained
*S* = 0.95	*w* = 1/[σ^2^(*F*_o_^2^) + (0.0597*P*)^2^ + 2.5255*P*] where *P* = (*F*_o_^2^ + 2*F*_c_^2^)/3
1844 reflections	(Δ/σ)_max_ < 0.001
164 parameters	Δρ_max_ = 0.56 e Å^−^^3^
0 restraints	Δρ_min_ = −0.32 e Å^−^^3^

### Special details

**Table:**

Geometry. All s.u.'s (except the s.u. in the dihedral angle between two l.s. planes) are estimated using the full covariance matrix. The cell s.u.'s are taken into account individually in the estimation of s.u.'s in distances, angles and torsion angles; correlations between s.u.'s in cell parameters are only used when they are defined by crystal symmetry. An approximate (isotropic) treatment of cell s.u.'s is used for estimating s.u.'s involving l.s. planes.
Refinement. Refinement of *F*^2^ against ALL reflections. The weighted *R*-factor *wR* and goodness of fit *S* are based on *F*^2^, conventional *R*-factors *R* are based on *F*, with *F* set to zero for negative *F*^2^. The threshold expression of *F*^2^ > 2σ(*F*^2^) is used only for calculating *R*-factors(gt) *etc*. and is not relevant to the choice of reflections for refinement. *R*-factors based on *F*^2^ are statistically about twice as large as those based on *F*, and *R*- factors based on ALL data will be even larger.

### Fractional atomic coordinates and isotropic or equivalent isotropic displacement parameters (Å^2^)

**Table:**

	*x*	*y*	*z*	*U*_iso_*/*U*_eq_	Occ. (<1)
N1	0.83007 (18)	0.08322 (16)	0.36167 (7)	0.0175 (4)	
N2	0.78338 (17)	−0.13986 (16)	0.35704 (7)	0.0175 (4)	
H2	0.7371	−0.2172	0.3616	0.021*	
Cl1	0.14851 (5)	0.07660 (6)	0.47297 (3)	0.0291 (2)	
C1	1.0106 (2)	−0.2127 (2)	0.30462 (9)	0.0198 (5)	
H1	0.9910	−0.3073	0.3034	0.024*	
C2	1.1337 (2)	−0.1595 (2)	0.28016 (9)	0.0213 (5)	
H2A	1.1994	−0.2183	0.2609	0.026*	
C3	1.1646 (2)	−0.0202 (2)	0.28307 (9)	0.0222 (5)	
H3	1.2509	0.0132	0.2659	0.027*	
C4	1.0721 (2)	0.0692 (2)	0.31040 (9)	0.0207 (5)	
H4	1.0945	0.1632	0.3128	0.025*	
C5	0.9449 (2)	0.01777 (19)	0.33442 (8)	0.0168 (4)	
C6	0.9160 (2)	−0.1225 (2)	0.33116 (8)	0.0172 (4)	
C7	0.7367 (2)	−0.01515 (19)	0.37430 (8)	0.0161 (4)	
C8	0.5944 (2)	0.0055 (2)	0.40055 (9)	0.0175 (4)	
C9	0.5678 (2)	0.1165 (2)	0.43598 (9)	0.0182 (4)	
H9	0.6435	0.1779	0.4454	0.022*	
C11	0.3186 (2)	0.0478 (2)	0.44523 (9)	0.0214 (5)	
C13	0.4824 (2)	−0.0846 (2)	0.38887 (9)	0.0223 (5)	
H13	0.5008	−0.1617	0.3653	0.027*	
C12	0.3456 (2)	−0.0646 (2)	0.41073 (9)	0.0234 (5)	
H12	0.2701	−0.1272	0.4023	0.028*	0.843 (4)
F1A	0.2476 (9)	−0.1424 (8)	0.4043 (4)	0.031 (3)	0.157 (4)
C10	0.4302 (2)	0.13632 (19)	0.45723 (9)	0.0187 (4)	
H10	0.4116	0.2133	0.4809	0.022*	0.157 (4)
F1	0.40066 (15)	0.24197 (14)	0.49152 (6)	0.0258 (5)	0.843 (4)

### Atomic displacement parameters (Å^2^)

**Table:**

	*U*^11^	*U*^22^	*U*^33^	*U*^12^	*U*^13^	*U*^23^
N1	0.0168 (8)	0.0154 (9)	0.0204 (9)	0.0005 (7)	0.0003 (7)	−0.0003 (7)
N2	0.0177 (9)	0.0122 (8)	0.0227 (9)	−0.0010 (7)	0.0020 (7)	0.0006 (7)
Cl1	0.0184 (3)	0.0294 (3)	0.0395 (4)	0.0006 (2)	0.0088 (2)	−0.0051 (2)
C1	0.0235 (11)	0.0152 (10)	0.0208 (10)	0.0036 (8)	0.0000 (9)	0.0009 (8)
C2	0.0229 (11)	0.0212 (11)	0.0199 (11)	0.0070 (9)	0.0007 (9)	−0.0001 (9)
C3	0.0184 (10)	0.0242 (11)	0.0239 (11)	0.0005 (9)	0.0029 (9)	0.0024 (9)
C4	0.0194 (10)	0.0162 (10)	0.0265 (11)	−0.0016 (8)	0.0000 (9)	0.0013 (8)
C5	0.0175 (10)	0.0159 (10)	0.0168 (10)	0.0026 (8)	−0.0007 (8)	0.0008 (8)
C6	0.0176 (10)	0.0182 (10)	0.0157 (10)	0.0018 (8)	−0.0011 (8)	0.0013 (8)
C7	0.0178 (10)	0.0146 (10)	0.0158 (10)	0.0026 (8)	−0.0043 (8)	−0.0017 (8)
C8	0.0190 (10)	0.0154 (9)	0.0180 (10)	0.0016 (8)	0.0000 (8)	0.0018 (8)
C9	0.0184 (10)	0.0157 (9)	0.0206 (10)	0.0002 (8)	0.0002 (8)	0.0023 (8)
C11	0.0189 (10)	0.0229 (11)	0.0226 (11)	0.0019 (9)	0.0030 (9)	0.0037 (9)
C13	0.0226 (11)	0.0185 (10)	0.0257 (12)	−0.0003 (9)	0.0011 (9)	−0.0034 (8)
C12	0.0202 (11)	0.0227 (11)	0.0273 (12)	−0.0027 (9)	0.0005 (9)	−0.0022 (9)
F1A	0.025 (4)	0.029 (5)	0.037 (5)	−0.006 (4)	0.001 (4)	−0.008 (4)
C10	0.0234 (11)	0.0135 (9)	0.0193 (10)	0.0039 (8)	0.0016 (8)	−0.0010 (8)
F1	0.0204 (8)	0.0207 (8)	0.0364 (9)	−0.0002 (6)	0.0054 (6)	−0.0126 (6)

### Geometric parameters (Å, º)

**Table:**

N1—C7	1.329 (3)	C5—C6	1.409 (3)
N1—C5	1.392 (3)	C7—C8	1.461 (3)
N2—C7	1.362 (3)	C8—C9	1.388 (3)
N2—C6	1.374 (3)	C8—C13	1.389 (3)
N2—H2	0.8800	C9—C10	1.376 (3)
Cl1—C11	1.719 (2)	C9—H9	0.9500
C1—C2	1.373 (3)	C11—C10	1.378 (3)
C1—C6	1.389 (3)	C11—C12	1.387 (3)
C1—H1	0.9500	C13—C12	1.375 (3)
C2—C3	1.403 (3)	C13—H13	0.9500
C2—H2A	0.9500	C12—F1A	1.195 (8)
C3—C4	1.380 (3)	C12—H12	0.9500
C3—H3	0.9500	C10—F1	1.337 (2)
C4—C5	1.394 (3)	C10—H10	0.9500
C4—H4	0.9500		
			
C7—N1—C5	104.82 (16)	N2—C7—C8	122.16 (18)
C7—N2—C6	107.33 (16)	C9—C8—C13	119.13 (19)
C7—N2—H2	126.3	C9—C8—C7	120.94 (18)
C6—N2—H2	126.3	C13—C8—C7	119.91 (18)
C2—C1—C6	117.30 (19)	C10—C9—C8	119.06 (19)
C2—C1—H1	121.4	C10—C9—H9	120.5
C6—C1—H1	121.4	C8—C9—H9	120.5
C1—C2—C3	121.41 (19)	C10—C11—C12	119.13 (19)
C1—C2—H2A	119.3	C10—C11—Cl1	120.18 (16)
C3—C2—H2A	119.3	C12—C11—Cl1	120.69 (17)
C4—C3—C2	121.40 (19)	C12—C13—C8	121.35 (19)
C4—C3—H3	119.3	C12—C13—H13	119.3
C2—C3—H3	119.3	C8—C13—H13	119.3
C3—C4—C5	118.11 (19)	F1A—C12—C13	123.9 (4)
C3—C4—H4	120.9	F1A—C12—C11	116.6 (4)
C5—C4—H4	120.9	C13—C12—C11	119.4 (2)
N1—C5—C4	130.80 (18)	C13—C12—H12	120.3
N1—C5—C6	109.54 (17)	C11—C12—H12	120.3
C4—C5—C6	119.63 (18)	F1—C10—C9	120.70 (18)
N2—C6—C1	132.36 (19)	F1—C10—C11	117.39 (18)
N2—C6—C5	105.50 (17)	C9—C10—C11	121.90 (19)
C1—C6—C5	122.12 (18)	C9—C10—H10	119.0
N1—C7—N2	112.82 (17)	C11—C10—H10	119.0
N1—C7—C8	124.92 (17)		
			
C6—C1—C2—C3	1.5 (3)	N2—C7—C8—C9	156.49 (19)
C1—C2—C3—C4	−0.3 (3)	N1—C7—C8—C13	150.8 (2)
C2—C3—C4—C5	−1.1 (3)	N2—C7—C8—C13	−25.3 (3)
C7—N1—C5—C4	178.2 (2)	C13—C8—C9—C10	−1.8 (3)
C7—N1—C5—C6	0.2 (2)	C7—C8—C9—C10	176.41 (18)
C3—C4—C5—N1	−176.6 (2)	C9—C8—C13—C12	1.3 (3)
C3—C4—C5—C6	1.2 (3)	C7—C8—C13—C12	−176.97 (19)
C7—N2—C6—C1	−178.1 (2)	C8—C13—C12—F1A	−176.2 (5)
C7—N2—C6—C5	0.3 (2)	C8—C13—C12—C11	−0.1 (3)
C2—C1—C6—N2	176.80 (19)	C10—C11—C12—F1A	175.8 (5)
C2—C1—C6—C5	−1.4 (3)	Cl1—C11—C12—F1A	−4.0 (5)
N1—C5—C6—N2	−0.3 (2)	C10—C11—C12—C13	−0.6 (3)
C4—C5—C6—N2	−178.58 (17)	Cl1—C11—C12—C13	179.62 (17)
N1—C5—C6—C1	178.26 (18)	C8—C9—C10—F1	179.91 (18)
C4—C5—C6—C1	0.0 (3)	C8—C9—C10—C11	1.2 (3)
C5—N1—C7—N2	0.0 (2)	C12—C11—C10—F1	−178.73 (18)
C5—N1—C7—C8	−176.36 (18)	Cl1—C11—C10—F1	1.1 (3)
C6—N2—C7—N1	−0.2 (2)	C12—C11—C10—C9	0.0 (3)
C6—N2—C7—C8	176.25 (17)	Cl1—C11—C10—C9	179.80 (16)
N1—C7—C8—C9	−27.5 (3)		

### Hydrogen-bond geometry (Å, º)

Cg is the centroid of the N1/C5/C6/N2/C7 ring.

**Table:**

*D*—H···*A*	*D*—H	H···*A*	*D*···*A*	*D*—H···*A*
N2—H2···N1^i^	0.88	2.06	2.924 (1)	166
C3—H3···*Cg*^ii^	0.95	2.92	3.700 (3)	140

Symmetry codes: (i) −*x*+3/2, *y*−1/2, *z*; (ii) *x*+1/2, *y*, −*z*+1/2.
