# Supplementary figures and images for: Crystal structure of 2-(4-chloro-3-fluoro­phen­yl)-1H-benzimidazole
Source: Acta Crystallogr E Crystallogr Commun. 2015 May 9;71(Pt 6):o387–8. doi: 10.1107/S2056989015008683 (PMC4459371; doi:10.1107/S2056989015008683)

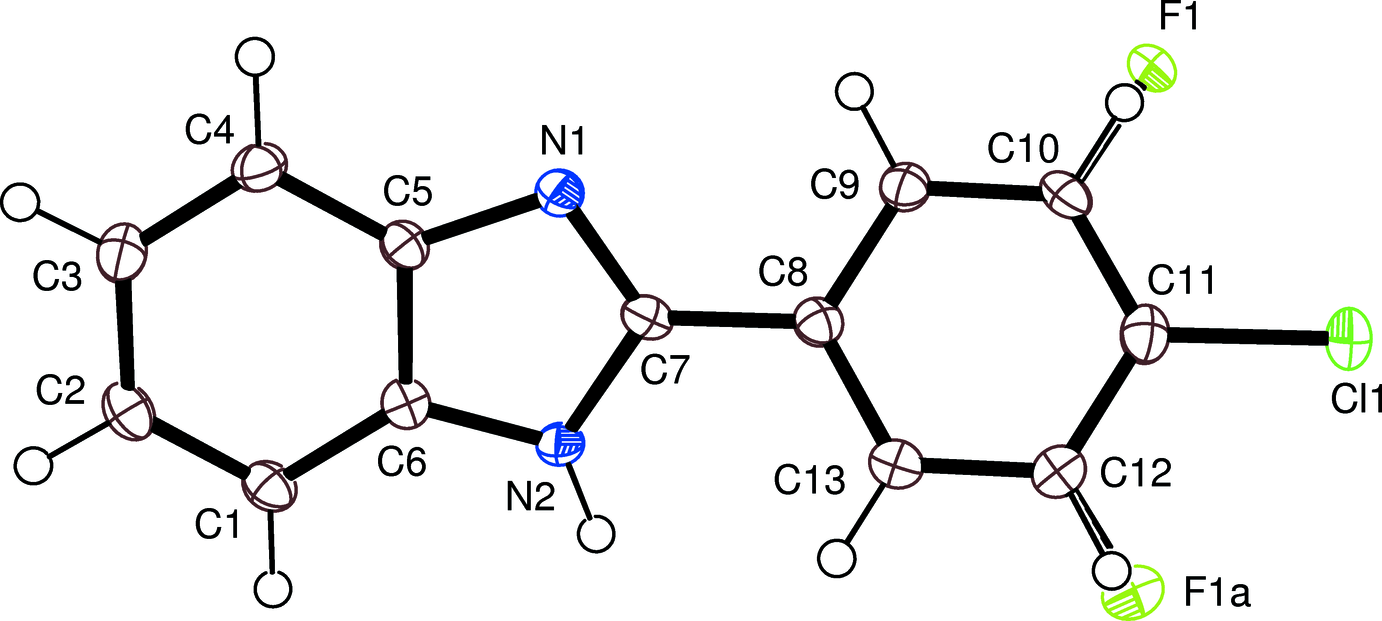

Supplement: Supplementary file 4 [file e-71-0o387-fig1.tif]

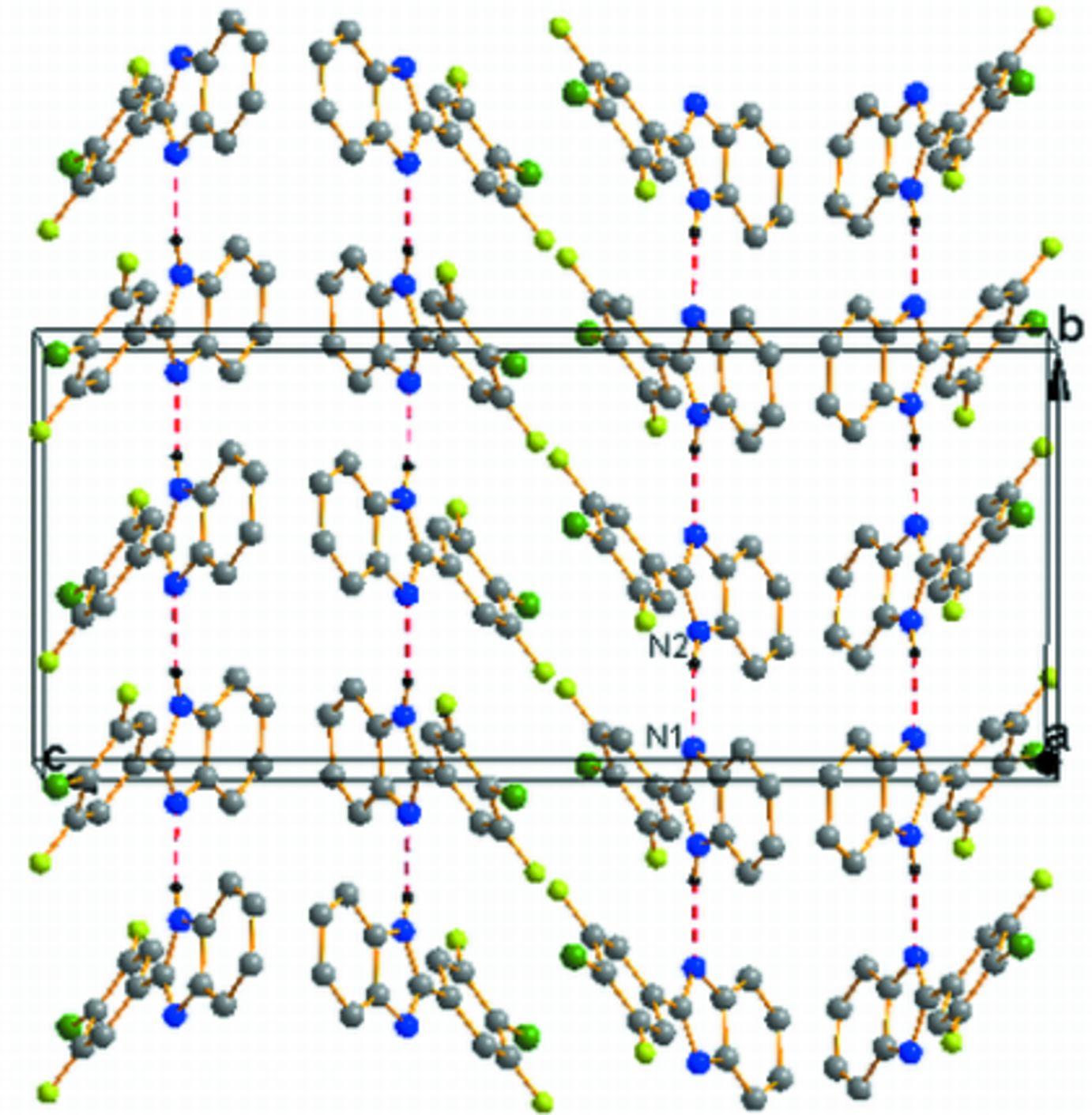

Supplement: Supplementary file 5 [file e-71-0o387-fig2.tif]

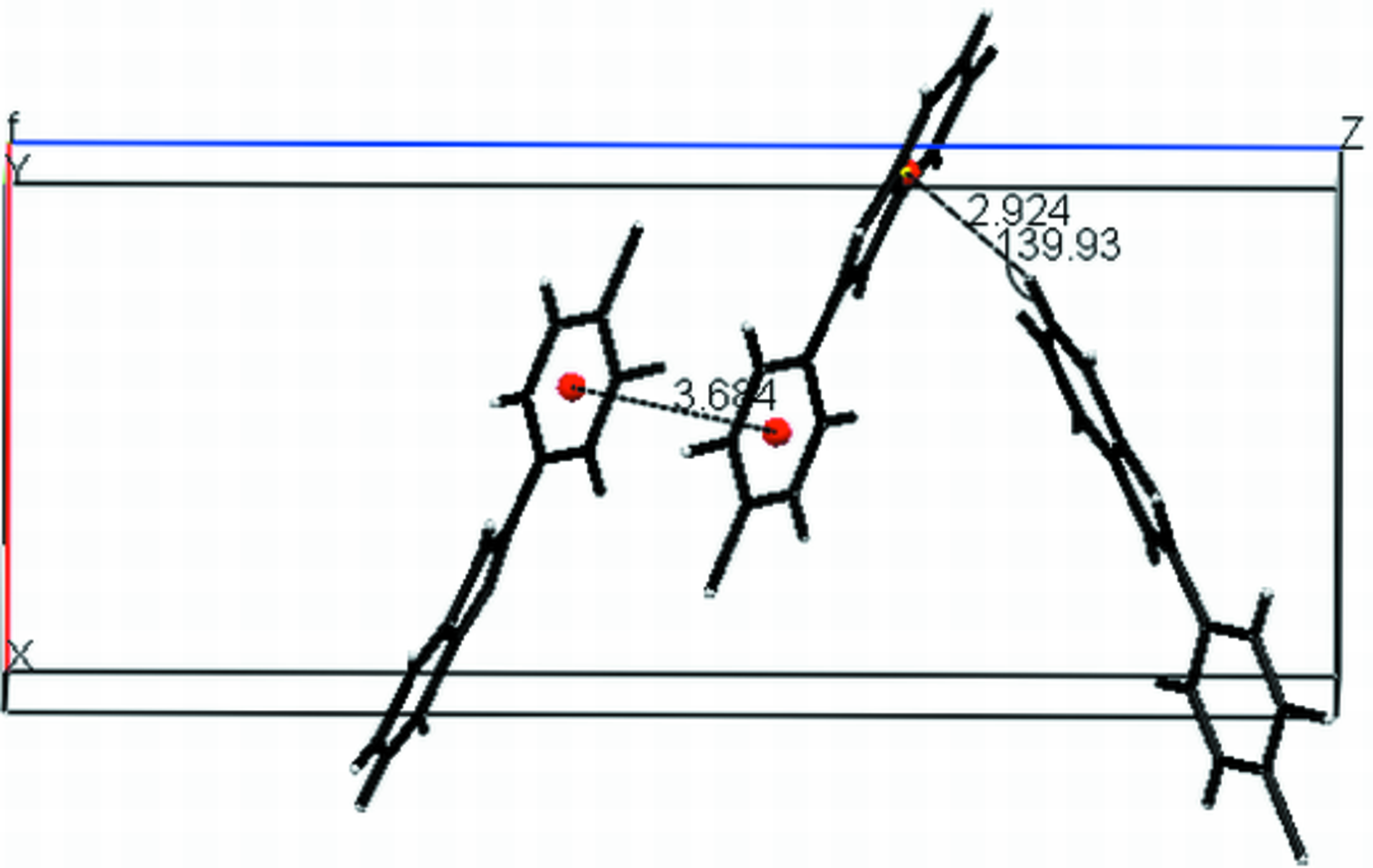

Supplement: Supplementary file 6 [file e-71-0o387-fig3.tif]
